# Bullous Disseminated Herpes Zoster: An Atypical Presentation

**DOI:** 10.7759/cureus.9293

**Published:** 2020-07-20

**Authors:** Tarang Jethwa, Raphael A. O Bertasi, Lisa Kieneker, Sweta Pattanaik, George Pujalte

**Affiliations:** 1 Family Medicine, Mayo Clinic, Jacksonville, USA; 2 Anesthesiology and Perioperative Medicine, Mayo Clinic, Jacksonville, USA

**Keywords:** herpes zoster, varicella zoster virus infection, atypical presentation, immunocompromised host

## Abstract

Herpes zoster is an infection resulting from the reactivation of dormant varicella zoster virus (VZV) in a posterior dorsal root ganglion. It affects 50% of immunocompromised patients and, when the viral infection persists, it can lead to a process known as disseminated varicella zoster virus (dVZV). Here we discuss a case of a bullous presentation of VZV with a rapid evolution of disseminated herpes zoster in an immunocompromised patient. Maintaining a broad differential diagnosis is necessary for early diagnosis and treatment of atypical presentations of herpes zoster, which is imperative to avoid increasing morbidity and mortality.

## Introduction

The reactivation of varicella zoster virus (VZV), which remains dormant in the sensory ganglia of the dorsal root, may cause a herpes zoster (HZ) infection [1]. It affects approximately 15% of immunocompetent patients, and the incidence can reach 50% in the immunocompromised population [2]. The main risk factor for this infection is advanced age due to senescence of the immune system. However, physical and psychological stressors, and immunosuppressed states, such as with cancer, autoimmune diseases, human immunodeficiency virus (HIV) infection, and solid organ and hematopoietic stem cell transplantations, should also be regarded as important triggers and risk factors [2].

The HZ infection is usually transient; it presents with less than 20 vesicles in the affected dermatome and can be treated with oral therapy. When the viral infection persists, it can lead to a process known as disseminated varicella zoster virus (dVZV), potentially leading to visceral involvement of the brain, liver, and lungs [3,4]. The immunocompromised population is more commonly affected and may have atypical presentations, which leads to challenges in prompt diagnosis and treatment [5].

Here we discuss a case of an atypical presentation of VZV with a rapid evolution of disseminated HZ in an immunocompromised patient. Diagnosis was made clinically and confirmed with polymerase chain reaction (PCR). Informed consent statement was obtained for this study. The atypical presentation, risk factors, and treatment are discussed. It is important for primary care physicians to maintain a high clinical suspicion for such a diagnosis, since it is essential for early diagnosis and treatment, in order to decrease mortality and morbidity.

## Case presentation

A 69-year-old female patient with a past medical history of lymphoma (initially treated with chemotherapy and radiation, and followed by an autologous hematopoietic stem cell transplant three years prior to this presentation, not currently on immunosuppressive therapy), chronic osteomyelitis with a stage IV decubitus ulcer on the right ischial tuberosity, recurrent urinary tract and Clostridioides difficile infections, diabetes mellitus type II, hyperlipidemia, hypertension, chronic kidney disease stage II, congestive heart failure, and chronic lower extremity radiculopathy presented to the emergency department (ED) secondary to a painless pruritic vesicular rash on her left shoulder and neck for one day that initially was treated with an “antibiotic cream,” the name of which she was unsure of.

In the ED, she reported that the rash had spread beyond the initial area and became painful. A diagnosis of HZ infection in the C6-C7 distribution was made, and she was discharged with a prescription of oral acyclovir 800 mg five times daily. Of note, the patient was not up to date with the VZV vaccine.

**Figure 1:**
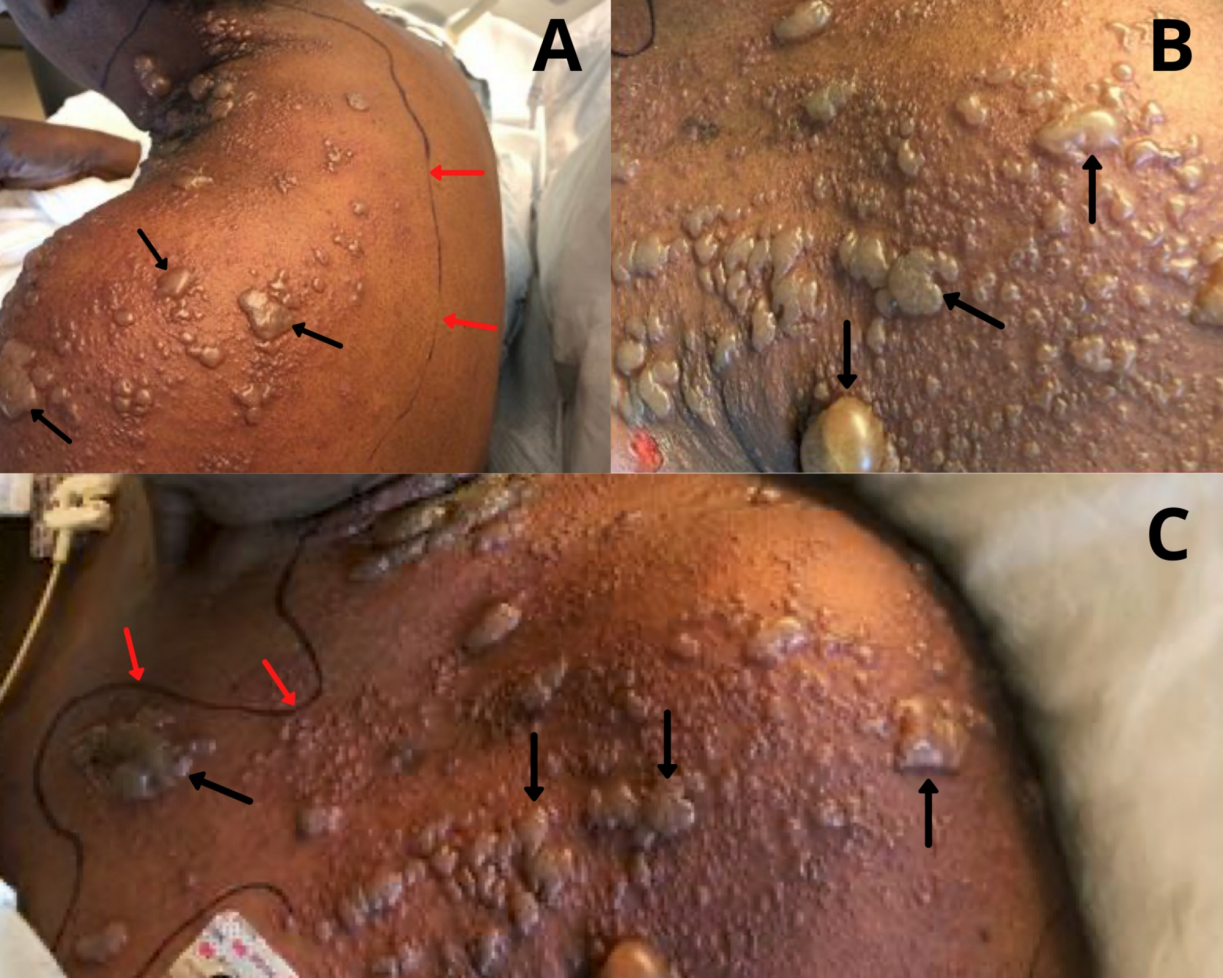
Bullous Presentation of Herpes Zoster. (A) Left latero-posterior view; (B, C) left latero-anterior view: Bullous vesicular lesions (black arrows) noted on admission with outlined area of erythema (red arrows).

After one day, she returned to the emergency room for altered mental status and the vesicular rash was noted to have spread over her chest, back, shoulder, and neck on the left side (Figure 1).

Physical exam was significant for fever (39.1°C) and a bullous vesicular rash in the left C5-T1 dermatomes with more than 20 lesions with serous drainage. Additionally, there were areas of open, ruptured bullae, with erythema and tenderness. Neurological examination revealed disorientation to time and place with bilateral lower extremity weakness and sensory loss. Intravenous (IV) vancomycin and cefepime, and acyclovir, were started for broad-spectrum coverage due to presumptive sepsis and failed to respond to oral acyclovir, concerning of central nervous system (CNS) infection due to encephalophatic presentation in the setting of bullous vesicular rash and chronic osteomyelitis with a stage IV decubitus ulcer on the right ischial tuberosity.

Labs, including blood and wound cultures, and a head CT were obtained. PCR taken from three vesicles in the left shoulder area was positive for VZV, and CT of the head had normal results. Lumbar puncture was not done due to patient refusal, and blood cultures were negative with initial leukocytes of 5,400/μL.

The patient was diagnosed with disseminated HZ infection, and antibiotics were discontinued after negative blood cultures. After three days of IV acyclovir, he was transitioned to oral acyclovir, which was continued for a total of three weeks. At the follow-up appointment five days after discharge, the lesions had crusted over; acyclovir ointment was prescribed for off-label use to help with pain and itching.

## Discussion

HZ is a transient infection presenting as a painful vesicular rash with lesions <5 mm diameter limited to one dermatome, with no more than 20 lesions outside the affected area [6]. Disseminated VZV occurs when viremia persists, leading to more than 20 vesicles outside the initial dermatome, with increased probability of visceral involvement [2].

Besides older age and immunosuppression, another risk factor for the development of dVZV is delayed treatment onset [7]. Therefore, a high clinical suspicion is crucial for prompt diagnosis and treatment, since disseminated disease has a high mortality rate: 5%-15% in non-HIV immunocompromised patients and 26% in HIV-positive patients [3]. In this case, the patient did not have a known precipitating factor, showing the importance of older age and immunosuppresion as risk factors.

Atypical presentation of HZ may challenge the proper diagnosis leading to delayed treatment as it may resemble other disorders. Additionally, there are clinical variants of HZ, including hemorrhagic, necrotizing, and bullous types [6]. There is scant literature regarding the bullous presentation. Several adjacent vesicles (<5 mm diameter) from the typical localized presentation merge and form greater bullous (>5 mm) areas, which is the defining characteristic of this variety [8,9].

To the best of our knowledge, the first case of bullous HZ was described in 2000 by Veraldi et al. [10]. They described a case of a 56-year-old immunocompetent women with bullous lesions in the submammary fold, which was treated with oral valacyclovir three grams daily for seven days. Similarly, bullous VZV was also diagnosed with PCR test in a three-year old child with leukemia, involving the first, second, and third fingers of right hand (compatibles to C6-C8 dermatomes), treated with IV acyclovir 500 mg three times a day [9]. Another study reported its occurrence in a 13-year-old girl with chronic glucocorticoid use involving the right neck (C2-C4 dermatomes) treated with foscarnet due to facial angioedema related to acyclovir initially administrated [6].

In dVZV with a bullous presentation, the lesions are outside the affected initial dermatome; it may resemble other bullous disorders, leading to a broader differential diagnosis including, but not limited to, infections (viral, such as with the herpes simplex virus, and bacterial, such as with staphylococcal scalded skin syndrome and bullous impetigo), trauma, allergy, phototoxicity, and immune-mediated diseases (bullous pemphigoid, bullous lupus erythematosus, pemphigus, etc.) [6]. HZ must be considered as a differential diagnosis when an immunocompromised elderly patient with multiple comorbidities presents with bullous skin lesions, regardless of the involvement of more than one specific dermatome, as seen in this case.

The treatment of choice for uncomplicated localized HZ is oral acyclovir 800 mg five times daily, valacyclovir 1,000 mg three times a day, or famciclovir 500 mg three times a day, for seven days [11]. If the initial treatment is delayed, there is an increased risk of the development of dVZV, for which treatment is IV acyclovir 10-15 mg/kg three times a day for 14 days [12].

In this case, our patient failed initial oral treatment and had rapid evolution, within 24 hours, of the primary vesicular rash to a bullous presentation, expanding to C5-T1 dermatomes. The early diagnosis and treatment with IV acyclovir led to improvement of the lesions and resolution of the neurological symptoms.

## Conclusions

A broad differential diagnosis and clinical acumen is necessary for diagnosing atypical presentations of HZ. The bullous presentation of the dVZV infection may resemble other disorders. Prompt diagnosis and treatment is imperative to avoid increasing morbidity and mortality. Uncomplicated HZ can be managed with oral treatment; however, those who fail it and progress to a disseminated disease should be hospitalized for IV therapy. 
